# Anti-Tumor Effects of Low Dose Zoledronate on Lung Cancer-Induced Spine Metastasis

**DOI:** 10.3390/jcm8081212

**Published:** 2019-08-14

**Authors:** Elie Akoury, Ana Sofia Ramirez Garcia Luna, Pouyan Ahangar, Xiaoya Gao, Pylyp Zolotarov, Michael H. Weber, Derek H. Rosenzweig

**Affiliations:** 1 Department of Surgery, Division of Orthopaedic Surgery, McGill University and the Research Institute of the McGill University Health Centre, Injury Repair & Recovery program, Montreal, QC H3G 1A4, Canada; 2 Medical Faculty Mannheim, Heidelberg University, D-68167 Mannheim, Germany; 3 Department of Pathology, McGill University Health Centre, Montreal, QC H4A 3J1, Canada

**Keywords:** spinal bone metastasis, lung cancer, zoledronate, low dose treatment

## Abstract

Zoledronate (Zol) is an anti-resorptive/tumoral agent used for the treatment of many cancers including spinal bone metastasis. High systemic administration of a single dose is now the standard clinical care, yet it has been associated with several side effects. Here, we aimed to evaluate the effects of lower doses Zol on lung cancer and lung cancer-induced bone metastasis cells over a longer time period. Human lung cancer (HCC827) and three bone metastases secondary to lung cancer (BML1, BML3 and BML4) cells were treated with Zol at 1, 3 and 10 µM for 7 days and then assessed for cell proliferation, migration, invasion and apoptosis. Low Zol treatment significantly decreased cell proliferation (1, 3 and 10 µM), migration (3 and 10 µM) and invasion (10 µM) while increasing apoptosis (10 µM) in lung cancer and metastatic cells. Our data exploits the potential of using low doses Zol for longer treatment periods and reinforces this approach as a new therapeutic regimen to impede the development of metastatic bone cancer while limiting severe side effects following high doses of systemic drug treatment.

## 1. Introduction

Lung cancer is one of the leading causes of cancer-related death worldwide [1,2]. In 2018, it was estimated that this disease will be responsible for 234,030 new cases and 154,050 deaths in the United States [3]. Tobacco smoking is the most significant risk factor for developing lung cancer [3,4]. Other risk factors include secondhand smoking, prior radiation exposure either for therapy or diagnostic imaging and exposure to environmental carcinogens, organic metals and chemicals [3,5,6,7,8]. Although smoking cessation is key in the fight against lung cancer, ex-smokers continue to be at increased risk of developing lung cancer during their lifetime [3].

In general, lung cancer can be classified histologically in two categories. The more common heterogeneous group called non-small cell lung cancer (NSCLC) tends to respond poorly to chemotherapy [9] and radiation therapy [3]. This group encompasses three major subcategories: adenocarcinoma, squamous cell carcinoma, and large cell carcinoma. Another group named small cell lung cancer (SCLC), also known as oat-cell carcinoma, is less frequent and characterized by a high proliferation rate, early metastasis [10] and sensitivity to combined chemotherapy and radiation therapy in limited-stage carcinoma cases [11,12]. Bone is known to be the second most common site of metastatic spread of lung cancer [13]. Metastatic disease to the bone compromises the structural integrity of the bone and significantly influences the quality of life of patients by causing pain or decreasing function [14]. Hence, the size of bone metastasis lesions positively correlates with the risk of sustaining a pathological fracture in the affected bone [14]. Determining new ways to reduce the growth or spreading of bone metastasis is of high importance since patients on palliative care are living longer [15].

Current treatment modalities for bone metastasis include tumor resection, often followed by allograft bone reconstruction, radiation therapy, chemotherapy and immunotherapy. The use of bisphosphonates is another treatment approach for patients with bone metastasis secondary to lung cancer [16,17], and is often given as an adjunct therapy. Bisphosphonates are a heterogenous group of medications with roles in inhibiting osteoclast-mediated bone resorption [18] and are widely used in the treatment of bone fragility secondary to osteoporosis [19] and other bone disorders such as osteogenesis imperfecta [20]. Investigations have proven the clinical benefit of bisphosphonates as an anti-resorptive agent in patients with various types of cancers, specifically in reducing the number of patients sustaining a skeletal related event by 30–50% [14,21]. As a result, the use of bisphosphonates has become a standard of care for the treatment and prevention of pathologic fractures secondary to bone metastases due to multiple myeloma and breast cancer [22]. Zoledronate is a third-generation nitrogen containing bisphosphonate and acts by disrupting the mevalonate pathway which ultimately results in osteoclast apoptosis [18]. Additionally, our work and that of others have shown the ability of Zol to exert direct anti-tumor function [23,24,25,26].

Although clinically effective, systemic Zol delivery is associated with several side effects that range from generalized musculoskeletal pain and aches to osteonecrosis of the jaw and ocular inflammation [27,28]. Therefore, local delivery of bisphosphonates at the site of the metastasis could be of great potential. For decades, researchers have been exploring different approaches to locally deliver bisphosphonates for various purposes including cancer treatment [29,30,31,32,33,34]. We previously demonstrated that local administration of Zol via percutaneous catherization into a xenograft mouse model resulted in improved bone quality at the metastatic site after tumor resection [33]. In the same study, we showed that local delivery of Zol increased apoptosis and decreased proliferation of metastatic cells in vivo [33]. Recently, we showed that low doses of Zol maintain their antitumor activity against prostate and bone metastatic tumor cells secondary to prostate cancer [26].

Herein, we demonstrate that low dose Zol impairs the ability of the lung cancer cell line and spine metastasis cells of lung cancer origin to proliferate, migrate and invade 3D matrix and enhances cell apoptosis. To our knowledge this is the first study to explore the application of low dose Zol treatment directly for longer term on lung cancer cell lines and lung cancer-induced bone metastasis cells in vitro. 

## 2. Results

### 2.1. Analysis of Lung Cancer-Induced Bone Metastasis Tissues

**Figure 1:**
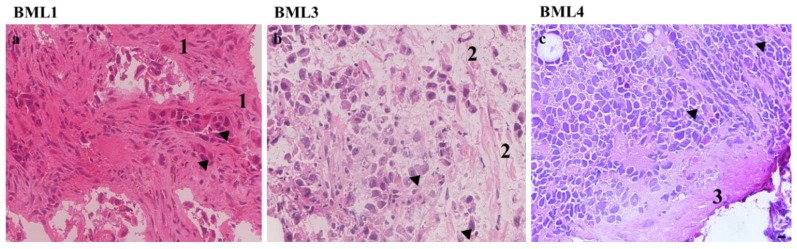
Histology hematoxylin–eosin (H&E) staining images of the three bone metastasis biopsies secondary to lung cancer (BML1, BML3 and BML4). (**a**) BML1, Non-small lung cancer cells, **1** designates abundant desmoplastic stroma. (**b**) BML3, non-small lung cancer cells, **2** edematous stroma. (**c**) BML4, small lung cancer cells, **3** Scant fibrous stroma. Black arrow heads indicate epithelial tumor cells with hyperchromatic pleomorphic nuclei and high nuclei/cytoplasm ratio. Scale bar 10 µM.

Tumor samples collected from patients undergoing surgery were analyzed at both the morphological and immunohistochemical levels. The information presented below was retrieved from the hospital pathological reports (immunomarkers) or provided by a second pathologist (morphology) (Figure 1).

BML1: H&E stained sections from the BML1 biopsies showed the presence of malignant epithelial cells forming glands. Upon immunohistochemical analysis with biomarkers, this tumor was found to be positive for CKAE1/3 (Cytokeratin AE1/3 used to confirm the epithelial origin), CK7 (Cytokeratin 7 and 20 were used to confirm the epithelial origin and define primary site; lung adenocarcinomas are usually cytokeratin 7 positive and cytokeratin 20 negative) and TTF1 (Thyroid transcription factor 1 and Napsin A were used to confirm the diagnosis of lung adenocarcinoma) whereas it was negative for CK20, Napsin A, Prostein and PSA (Prostein and Prostate specific antigen were used to exclude a possible metastatic prostate adenocarcinoma as prostate adenocarcinoma is one of the most common bone metastases in male patient). Therefore, this tumor was classified as non-small cell lung carcinoma (NSCLC) with a subclassification of adenocarcinoma (Figure 1a).

BML3: Following histology analysis, this tumor manifested clusters of malignant epithelial cells which suggests its classification as carcinoma. Further immunohistochemical profiling showed that this BML3 was positive for CEA (Carcinoembryonic antigen usually positive in adenocarcinoma) and CK7 but was negative for TTF1, Napsin A and PSA. This pattern of immunostaining, along with the fact that the tumor did not have classic morphology of adenocarcinoma and was not tested for squamous carcinoma markers, therefore classified this tumor as non-small cell lung carcinoma (NSCLC) not otherwise specified (Figure 1b).

BML4: Histology revealed the presence of malignant pleomorphic hyperchromatic cells with nuclear molding, suggesting the classification of BML4 as small cell carcinoma. Immunohistochemical profile demonstrated that BML4 was positive for CKAE1/3, Synaptophysin, Chromogranin and CD56 (Synaptophysin, Chromogranin and CD56 were used to confirm neuroendocrine differentiation). However, this tumor was negative for CK7, CK20, CK5/6, p40 (Cytokeratin 5/6 and p40 were used to exclude squamous differentiation), S100 (S100 was used to exclude possible metastatic melanoma due to high grade tumor morphology) and CD45 (CD45 wasused to exclude hematopoietic origin as with this small blue cell morphology lymphoma was in the differential diagnosis). Altogether, this tumor was therefore classified as small cell lung carcinoma (SCLC) (Figure 1c).

### 2.2. Zoledronate Decreases Lung Cancer Cell Line Proliferation

**Figure 2:**
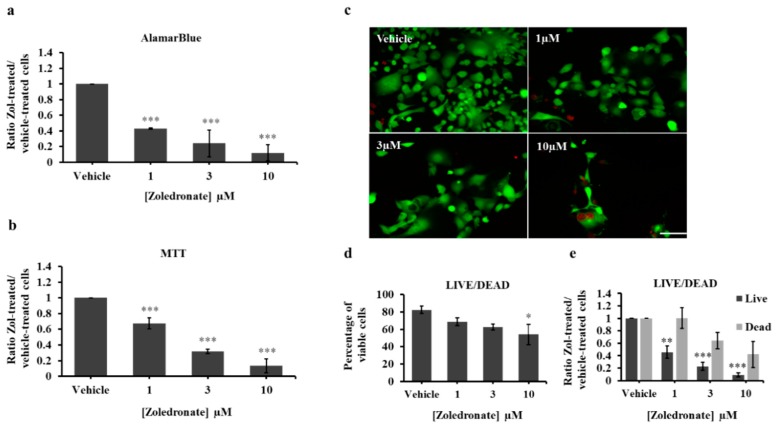
AlamarBlue (**a**) and MTT (**b**) assays of HCC827 cells treated with vehicle (PBS1x) or Zol 1 µM, 3µM and 10 µM for 7 days in 1% serum conditions. The histograms in (a) and (b) represent the ratio of drug-treated cells divided by vehicle-treated cells (PBS1x) in three independent experiments. (**c**) representative photos of Live/Dead assay carried out on HCC827 following vehicle or Zol treatment at different concentrations. Live cells are in green and dead cells are in red. Scale bar 250 µm. (**d**) Percentage of viable cells [number of live cells/(number of live cells + number of dead cells) × 100]. (**e**) ratio of live cells or dead cells in vehicle or Zol-treated conditions. Significantly different from control * *p* < 0.05, ** *p* < 0.01 and *** *p* < 0.001.

We previously demonstrated that low doses Zol (≤10 μM) inhibit cell proliferation of prostate cancer and prostate cancer-induced bone metastasis cells in vitro over a course of 7 days [26]. Here, we tested the efficacy of the same low range of Zol concentrations (1, 3 and 10 μM) and incubation conditions on a commercially available lung cancer cell line HCC827 before assessing cell proliferation. Using alamarBlue® assay, treated HCC827 showed a significant, dose dependent decrease in cell proliferation (Figure 2a). Zol-treated cells at 10 μM and 3 μM showed the largest decrease averaging 89% ± 10.4% (*p* value < 0.001) and 76% ± 17% (*p* value < 0.001) respectively, compared to controls following a one-week treatment. Additionally, 1 μM revealed a significant, albeit less, decrease in proliferation averaging 57% ± 0.95% (*p* value < 0.001). MTT Vybrant assay also revealed a significant reduction in cell proliferation with 10 μM showing the highest effect (86.6% ± 8.8%, *p* value < 0.001), and to a lesser degree 3 μM (68.1% ± 2.85%, *p* value < 0.001) and 1 μM (32.7% ± 7.09%, *p* value < 0.001) (Figure 2b). Further validation with Live/Dead assay demonstrated that the percent viable cells was significantly reduced in 10 μM (54% ± 11.58%, *p* value 0.049), but not in 1 μM (68.62% ± 4.28%, *p* value = 0.138) nor in 3 μM (62.46% ± 3.49%, *p* value = 0.065) as compared to vehicle-treated controls (82.4% ± 4.34%) (Figure 2c–e). Altogether, these data indicate that low dose Zol treatment impairs HCC827 cell proliferation in vitro over 7 days.

### 2.3. Zoledronate Decreases the Proliferation of Lung Cancer-Induced Bone Metastasis Cells

**Figure 3:**
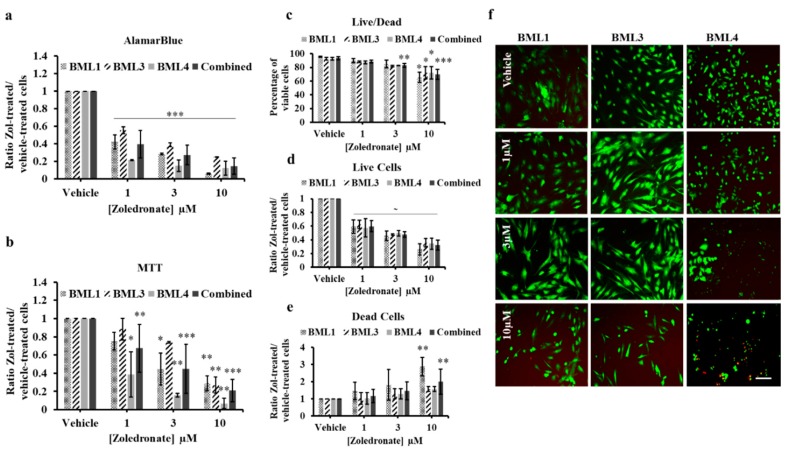
AlamarBlue (**a**) and MTT (**b**) assays of lung cancer-induced bone metastasis cells treated with vehicle (PBS1x) or Zol 1 µM, 3 µM and 10 µM for 7 days in 1% serum conditions. The histograms in (a) and (b) represent the ratio of drug-treated cells divided by vehicle-treated cells (PBS1x) in three independent experiments. (**c**) Percentage of viable cells [number of live cells/(number of live cells + number of dead cells) × 100] and (**d**) ratio of live cells or (**e**) ratio of dead cells in vehicle or Zol-treated conditions of Live/Dead assay carried out on bone metastasis cells. (**f**) representative photos of Live/Dead assay following vehicle or Zol treatment at different concentrations. Live cells are in green and dead cells are in red. Scale bar 250 µM. Significantly different from control * *p* < 0.05, ** *p* < 0.01, *** *p* < 0.001, ^~^ *p* values indicated in the RESULTS text.

To determine the effect of Zol in a more clinical relevance, bone metastasis cells secondary to lung cancer were isolated from biopsies of three patients undergoing tumor resection (BML1, BML3 and BML4) before being treated with Zol. Using alamarBlue assay, we observed a significant, dose-dependent decrease in cell proliferation of the NSCLC lung-induced bone metastasis cells at 10 μM (BML1, 93.9% ± 0.71%, *p* value < 0.001; BML3, 75% ± 0.25%, *p* value < 0.001), 3 μM (BML1, 71.5% ± 0.76%, *p* value < 0.001; BML3, 61.1% ± 2.24%, *p* value < 0.001,) and 1 μM (BML1, 57.7% ± 7.9%, *p* value < 0.001; BML3, 44.6% ± 3.8%, *p* value < 0.001) compared to vehicle-treated cells (Figure 3a). On the other hand, SCLC (BML4) cells manifested a significant but dose-independent decrease at all concentrations (10 μM, 87.5% ± 7.8%, *p* value < 0.001; 3 μM, 84.9% ± 6.5%, *p* value < 0.001; 1 μM, 78.4% ± 0.6%, *p* value < 0.001). Combining the data of all three bone metastasis donors, we observed an overall statistically significant drop-off in proliferation at all treatment conditions compared to controls (10 μM, 85.4% ± 9.3%, *p* value < 0.001; 3 μM, 72.5% ± 11%, *p* value < 0.001; 1 μM, 60.2% ± 15.7%, *p* value < 0.001) (Figure 3a). MTT assay showed the same significant reduction in proliferation in all analyzed individual NSCLC and SCLC cells (Figure 3b): BML1 (10 μM, 71% ± 8.1%, *p* value = 0.0098 and 3 μM, 55% ± 17.6%, *p* value = 0.023), BML3 (10 μM, 73.2% ± 8.78%, *p* value = 0.0021) and BML4 (10 μM, 93.2% ± 5.6%, *p* value = 0.0063; 3 μM, 84.1% ± 1.8%, *p* value = 0.009 and 1 μM, 61.3% ± 24.6%, *p* value = 0.028) except for BML1 at 1 μM (25% ± 9.6%, *p* value = 0.24) and BML3 at 1 μM (11.6% ± 11.9%, *p* value = 0.48) and 3 μM (26.1% ± 0.87%, *p* value = 0.078). When all three BMLs were combined, the same significant decline in proliferation persisted in 10 μM (79.2% ± 12.4%, *p* value < 0.001), 3 μM (55.3% ± 27.1%, *p* value < 0.001) and 1 μM (32.6% ± 26.4%, *p* value = 0.007) as compared to the vehicle-treated conditions. Further validation with Live/Dead assay (Figure 2c–f) showed the same percent viable cells in all conditions except for Zol 10 µM which showed a significant decrease for both NSCLC cells (BML1, 65.72% ± 7.55%, *p* value = 0.012; BML3, 72.04% ± 9.02%, *p* value = 0.041) and SCLC cells (BML4, 72.04% ± 9.023%, *p* value = 0.043). Upon pooling BMLs data, the percent of viable cells was reduced in 3 μM (83% ± 2.9%, *p* value = 0.002) in addition to 10 μM (69.9% ± 7.3%, *p* value < 0.001) compared to controls. Altogether these data indicate that treatment with low doses Zol decreases proliferation of lung cancer-induced bone metastasis in vitro.

### 2.4. Zoledronate Increases Apoptosis of HCC827 and Lung Cancer-Induced Bone Metastasis Cells

**Figure 4:**
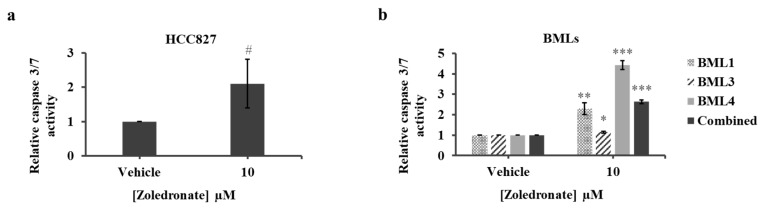
Caspase 3/7 activity assay of HCC827 (**a**) and lung cancer-induced bone metastasis cells (**b**) cells treated with vehicle (PBS1x) or Zol 10 µM for 7 days in 1% serum conditions. The histograms represent the ratio of drug-treated cells divided by vehicle-treated cells (PBS1x). Results are the mean ± SD of three independent experiments for HCC827 and bone metastasis cells. Marginally significant # *p* ≥ 0.05 and significantly different from control, * *p* < 0.05, ** *p* < 0.01 and *** *p* < 0.001.

Zoledronate treatment was previously shown to induce apoptosis in NSCLC and SCLC cells [35,36,37]. We therefore sought to investigate whether low Zol treatment exhibits an apoptotic property on cancer cells. We chose 10 μM among the tested range of Zol concentrations and treated lung cancer and lung cancer-induced bone metastasis cells for 7 days before caspase 3/7 activity was assessed. Treatment of HCC827 cells showed an approximately significant 2-fold increase in the caspase activity compared to controls (2.10 ± 0.7, *p* value = 0.0536) (Figure 4a). When assessing caspase activity of lung cancer-induced bone metastasis cells, caspase levels increased significantly in the treated BML1 (2.28 ± 0.28, *p* value = 0.0014), BML3 (1.15 ± 0.04, *p* value = 0.038) and the BML4 cells (4.43 ± 0.22, *p* value < 0.001) compared to vehicle-treated cells (Figure 4b). Combining the values of all BMLs revealed a significant increase (2.63 ± 0.09, *p* value < 0.001) compared to controls (Figure 4b). Since apoptosis could be triggered earlier, we quantified caspase3/7 activity following a 4-day treatment and observed a significant increase only in the combined BMLs. Altogether, these data indicate that treatment with low dose Zol (10 μM) increases apoptosis of primary lung cancer and lung cancer-induced bone metastasis in vitro.

### 2.5. Zoledronate Affects the Migration of HCC827 and Lung Cancer-Induced Bone Metastasis Cells

**Figure 5:**
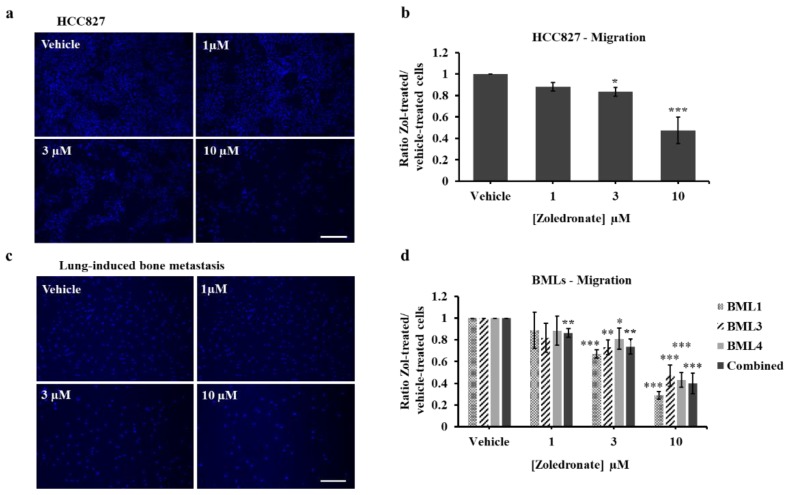
Migration (Transwell chamber cell assay) of HCC827 (**a**,**b**) and lung cancer-induced bone metastasis cells (**c**,**d**) treated with vehicle (PBS1x) or Zol 1 µM, 3µM and 10 µM for 7 days in 1% serum conditions. Representative images of HCC827 (**a**) and bone metastasis (BML3) (**c**) cells from vehicle or Zol-treated conditions. Scale bar 100 µM. The histograms represent the ratio of drug-treated cells divided by vehicle-treated cells (PBS1x) for HCC827 (**b**) and bone metastasis (**d**) cells. Results are the mean ± SD of three independent experiments for HCC827 and bone metastasis cells. Significantly different from control * *p* < 0.05, ** *p* < 0.01 and *** *p* < 0.001.

To test the effects of low dose Zol on HCC827 and lung cancer-induced bone metastasis cells on migration, a transwell chamber cell migration assay was performed. Compared to vehicle, we found that HCC827 migration from the upper side to the underside of the chamber was significantly decreased following treatment with 3 μM (23.5% ± 8.1%, *p* value = 0.019) and was more pronounced with 10 μM (55.6% ± 11.98%, *p* value < 0.001) (Figure 5a,b). To exclude any cell proliferation effect, we synchronized HCC827 cells in serum-free medium for 24 h and then switched to our experimental conditions. Following analysis, we observed the same significant decrease in migration for 3 μM- and 10 μM-treated cells as in unsynchronized-treated cells. We therefore carried out all subsequent migration analysis without cell synchronization. Treatment of lung cancer-induced bone metastasis cells with Zol also significantly decreased migration of cells with 3 µM for BML1 (32.8% ± 3.7%, *p* value < 0.001), BML3 (26.8% ± 6.7%, *p* value = 0.002) and BML4 (19% ± 9.7%, *p* value = 0.028) and to a higher extent with 10 µM for BML1 (71% ± 3.4%, *p* value < 0.001), BML3 (52.9% ± 9.8%, *p* value < 0.001) and BML4 (56.8% ± 6.7%, *p* value < 0.001) compared to controls (Figure 5c,d). When pooling the data of all BMLs, the impaired migration carried through at 10 μM (60.2% ± 9.5%, *p* value < 0.001), 3 μM (26.2% ± 6.9%, *p* value = 0.0028) and appeared at 1 μM (13.6% ± 3.91%, *p* value = 0.0038). Altogether, these data indicate that low dose Zol treatment of 3 μM and 10 μM over 7 days can effectively inhibit lung and metastatic tumor cell migration.

### 2.6. Zoledronate Affects the Invasion of HCC827 and Lung Cancer-Induced Bone Metastasis Cells

**Figure 6:**
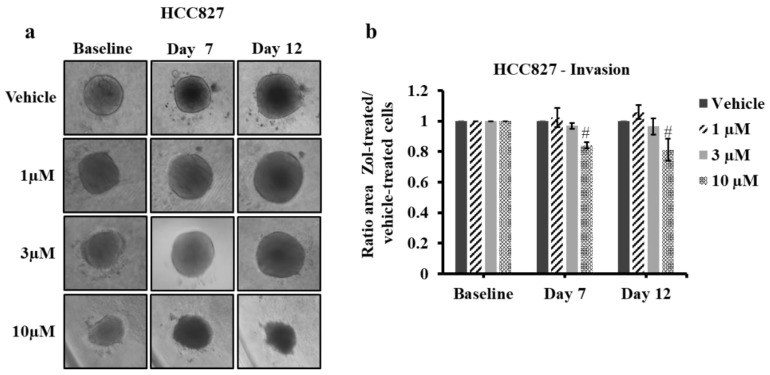
Spheroid invasion assay of HCC827 cells treated with vehicle (PBS1x) or Zol 1 µM, 3 µM and 10 µM for 7 and 12 days in 1% serum conditions. (**a**) Representative brightfield images of HCC827 spheroid invasion/growth within the invasion matrix from vehicle or Zol-treated conditions. (**b**) Histograms for invasion/growth in the matrix showing the mean of three independent experiments ± SD. Each condition (drug-treated cells or vehicle-treated cells) on each day was normalized to day 0 and then to vehicle. Close to be significantly different from control # *p* ≥ 0.05.

To test the effects of low dose Zol on spheroid growth and invasion, 3D matrix cultures were generated using HCC827 and the three lung cancer-induced bone metastasis cells and treated with Zol at 1, 3 and 10 µM for 7 and 12 days. HCC827 spheroids embedded in the surrounding matrix did not invade in either the drug-treated or untreated cells (Figure 6a). However, HCC827 cell expansion measured by the surface area was smaller, approaching a borderline statistical significance, when spheroids were treated with 10 µM Zol as compared to controls at both 7 days (16% ± 10.8%, *p* value = 0.05) and 12 days (18.8% ± 19.1%, *p* value = 0.07) (Figure 6a,b). Since cell invasion could be activated earlier, we assessed spheroid expansion following 1-day and 4-day treatments using the same range of Zol concentrations and observed no significant difference at all concentrations in the drug-treated HCC827 cells as compared to controls (Figure S1).

**Figure 7:**
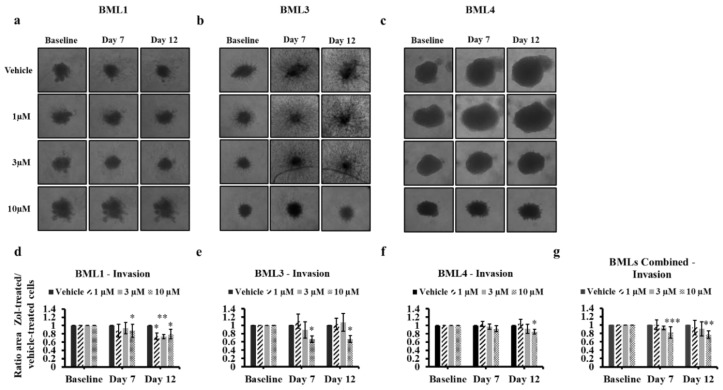
Spheroid invasion assay of lung cancer-induced bone metastasis cells treated with vehicle (PBS1x) or Zol 1 µM, 3 µM and 10 µM for 7 and 12 days in 1% serum conditions. Representative brightfield images of bone metastasis cell spheroid growth (**a**–**c**) within the invasion matrix from vehicle or Zol-treated conditions. Histograms for growth in the matrix (**d**–**g**) showing the ratio of drug-treated cells divided by vehicle-treated cells (PBS1x) ± SD for three independent experiments. (**g**) combined ratio for the three cell types of bone metastasis cells are presented. Each condition (drug-treated cells or vehicle-treated cells) on each day was normalized to day 0 and then to vehicle. Significantly different from control * *p* < 0.05, ** *p* < 0.01 and *** *p* < 0.001.

When assessing spheroid invasion of the lung cancer-induced bone metastasis cells following Zol treatment, these cells behaved differently. BML1 and BML3 spheroids were able to migrate out the spheroids and infiltrate into the surrounding matrix in vehicle- or Zol-treated conditions (Figure 7a,b,d,e). However, the spheroid surface area was significantly smaller over 12 days for BML1 at 10 µM (20.9% ± 11.3%, *p* value = 0.03), 3 µM (26.8% ± 4.9%, *p* value = 0.008) and 1 µM (25.7% ± 7.7, *p* value = 0.01) and for BML3 only at 10 µM (33.1% ± 8.4%, *p* value = 0.02). It is important to note that BML 1 and BML 3 spheroids at day 7 showed also significantly smaller surface area 12.7% ± 15.5%, *p* value = 0.01 and 33.2% ± 7.8%, *p* value = 0.01) respectively following treatment with 10 µM of Zol. For BML4, no cell invasion was observed in all conditions; however, the surface area of spheroids was significantly smaller only with 10 µM (15.3% ± 5.9%, *p* value = 0.046) following the 12-day treatment (Figure 7c,f). Combined values for all three BMLs revealed a significant inhibition of cell invasion that only prevailed at 10 µM for both day 7 (18% ± 13.3%, *p* value < 0.001) and day 12 (23% ± 9%, *p* value = 0.008) (Figure 7g). When cell invasion was checked at early time points in metastatic cells, a significant difference in spheroid surface area was only observed at 10 µM following a 1-day treatment in BML3 (12.2% ± 4.8%, *p* value = 0.04), BML4 (3.8% ± 1.3%, *p* value = 0.03) and combined BMLs (7.2% ± 4.4%, *p* value = 0.003) and following a 4-day treatment in BML3 (33.8% ± 7.6%, *p* value = 0.01), BML4 (7.7% ± 1.7%, *p* value = 0.01), and combined BMLs (17.4% ± 14.2%, *p* value = 0.009) (Figure S1).

These data indicate that within a more physiological 3D-matrix environment, treatment with low dose Zol (10 µM) over 12 days can significantly reduce HCC827 and NSCLC/SCLC lung-induced bone metastasis spheroid growth and individual cell outgrowth.

## 3. Discussion

Over recent decades bisphosphonates, mostly Zol, have become a cornerstone for the treatment of solid tumors as well as bone metastasis secondary to a wide range of neoplasms of the breast, prostate and lung. The current regimen of Zol treatment for patients is a single 4 mg intravenous dose every 3–4 weeks. This standard treatment has been proven to reduce the risk of developing skeletal related events and delaying the onset of complications secondary to bone metastasis [38,39,40,41,42], while slowing down tumor growth in patients [23,24,25]. This high systemic dose, however, causes debilitating side effects ranging from flu-like symptoms to osteonecrosis of the jaw and renal toxicity [27]. In an attempt to circumvent these shortcomings, we tested for the first time a low range of Zol concentrations (1, 3 and 10 µM) in vitro over a 7-day treatment. This was performed under low serum conditions on NSCLC lung cancer cell line HCC827 and three lung cancer-induced bone metastasis cells, of which two arose from NSCLC cells (BML1 and BML3) and one originated from SCLC cells (BML4). We assessed several key steps of cancer growth and development including cell proliferation, migration and invasion [43,44] following drug treatment. Under these conditions, 1, 3 and 10 µM Zol significantly slows down the proliferation at a range of 20 to 80% for all cell types with BML4 having the highest sensitivity. These Zol doses were up to 100 times less than the concentrations previously used to decrease proliferation of human NSCLC including HCC827 and SCLC cell lines (in vitro for less than 5 days) [35,36,37,45]. In all these studies, the impaired proliferation was attributed either to an increase in cell apoptosis or to a disruption of cell cycle [35,36,37,45]. Moreover, it was suggested that bisphosphonates promote apoptosis in the bone-colonizing cancer cells as a result of restricting the supply of bone-stored growth factors that are crucial for the proliferation and survival of cancer cells following the inhibition of osteoclastic bone resorption [46,47,48,49,50,51]. These findings are in line with our data demonstrating the capability of low dose Zol (10 µM) and long-term treatment to trigger apoptosis and eventually decrease proliferation of the analyzed lung cancer and metastatic bone cells. 

When cell migration following Zol treatment was investigated, this cellular process was also significantly defective in a dose dependent fashion manifesting a decrease ranging from 20–70% at 3 µM and 10 µM in Zol-treated cells for NSCLC (HCC827), NSCLC bone metastasis (BML1, BML3), and SCLC cells (BML4). Again, this anti-migratory effect of Zol under our conditions was as adept as the one previously reported on five different lung cancer NSCLC cell lines (40–70%), where Zol treatment was proved to be effective only at high concentrations (≤100 μM) for 24 h [45]. The study of Kenessey et al. suggested that Zol inhibited the mevalonate pathway leading to the prevention of post-translational modification i.e prenylation of small G-proteins involved in motility signaling (such as Rho, Rac, Cdc42) and consequently resulted in an impaired migratory capacity [45]. Although the treatment and culture conditions are different in our study, such a suggestion seems conceivable at least to some extent for Zol-treated NSCLC cell line and NSCLC metastatic cells and perhaps for metastatic SCLC cells. However, further investigation is needed to elucidate the mechanism underlying inhibitory effects of Zol on migration for all the analyzed cells that stem from different origins. Another interesting aspect of the in vitro effects of Zol is reduction in cell invasion. We assessed 3D spheroid invasion on the lung cancer and bone metastasis cells. Treatment with 10 µM Zol blocked the 3D invasion/growth of all NSCLC (BML1, BML3 and HCC827) following 7 days and for all NSCLC and SCLC (BML4) cells following 12 days. When short-term Zol treatment was applied on the same cells, cell invasion was significantly impaired only in BML3 and BML4 at 10 µM condition. This indicates that treatment with low dose Zol (10 µM) for 1–4 days inhibits cell invasion/growth in some lung cancer-induced bone metastasis cells but not in lung cancer NSCLC (HCC827) cells and therefore is less effective than long-term treatment in hindering cell invasion of NSCLC and SCLC cells under our conditions. Moreover, it is noteworthy that 3 µM Zol, which significantly decreased cell proliferation and migration, did not affect the cell invasion/growth of any of the analyzed lung or metastatic cells in general for either short- or long-term treatment. On the other hand, a previous study by Futamura et al., demonstrated the Zol inhibition of invasion on two treated human lung cancer cell lines using a low dose of 5 µM, half of highest tested dose in our study [52]. However, the experimental conditions were quite different and not using a 3D microenvironment, thereby preventing a strong comparison to the study presented here.

Nevertheless, our results on long-term treatment and low doses Zol are in good agreement with our own data on prostate bone metastasis cells treated with Zol at the same range of concentrations under the same culture environment and therefore the same speculations could apply here [26]. Indeed, it is well established now that tumor growth conditions are not similar in 3D vs. 2D cultures [53], and tumor expansion in 3D culture is more resistant to drug treatment [54]. Therefore, higher doses of therapeutics are required to achieve inhibition/impairment of a cellular function in a specific experimental context. Mechanistic studies are also deemed necessary to understand the Zol anti-invasiveness effect on lung and bone metastasis cells of lung origin. Nevertheless, our data on lung and bone cancer cells support the idea that low dose Zol (10 µM) for 7–12 days is indeed able to decrease cancer cell invasion in 3D environment.

The single dose of zoledronate prescribed to patients would only maintain a peak serum concentration of 1–3 µM for few hours following systemic administration [55], since most of the Zol encompasses high affinity for mineralized bone and consequently settles to bone [56]. Therefore, the low doses of Zol (≤10 µM) tested here in vitro, if adopted into clinical practice, would certainly be insufficient to reach the tumor site and apply their anti-tumor activity. Local delivery exposure could therefore present an exciting potential to sustainably release low doses of pharmaceutical compounds with anti-tumor properties directly at the tumor site. Our previous work and that of others have provided promising therapeutic approaches for local drug delivery including the use of subcutaneous catheterization [33], nanoparticles carriers [57,58], bone cement [59] and nanohydroxyapatite scaffolds [60]. Our team recently developed 3D-printed nanoporous PORO Lay scaffolds that uptake and deliver low doses of doxorubucin [61] or Zol [34], which at the same time inhibited the proliferation of prostate cancer-induced bone metastasis cells in vitro. Future work is indeed needed to test the same 3D printed scaffold—Zol releasing technology for local delivery in vitro to lung tumors and metastatic bone cells and in vivo to xenograft and patient-derived xenograft animal models. These scaffolds could be further adjusted for highly tuned and controlled release and more accurate dosing by the application of surface coating with the use of nanoclay particles [62] chitosan [63], poly lactic acid (PLA) [64] or polycaprolactone (PCL) [65]. Also, one could attempt to explore the co-administration of these scaffolds with more than one drug or bioactive substance to achieve more impressive anti-tumor effects or additionally promoting normal bone healing and repair at the bone-tumor defect site following surgery. Moreover, these scaffolds can be designed, custom 3D printed and tailored to meet patients’ anatomical needs and consequently fit at the resected bone/tumor site. Although clinically relevant, the 3D printed biomaterials cannot be applied as a bone substitute to fill load-bearing defects of post-tumor resection. This is attributed to the fact that PORO Lay materials lack mechanical strength compared to bone [61] or other 3D printed materials like PLA or acrylonitrile butadiene styrene (ABS) [66]. To overcome this obstacle, PORO Lay materials impregnated with the drug could be co-printed to generate composite scaffolds with PLA, poly lactic co glycolic acid (PLGA) or PCL of adequate bone-like mechanical properties [64,65,67,68] providing a mechanically stable implant for reconstructive surgery post bone tumor resection.

In conclusion, we show that Zol treatment in vitro at low concentrations over 7 days decreases cell proliferation, migration and 3D invasion but increases apoptosis of the NSCLC lung cancer cell line (HCC827) and NSCLC/SCLC spine metastasis cells secondary to lung cancer. Our data highlights the anti-tumor properties of long treatment of low doses Zol on lung cancer and metastatic cells. Additionally, these results bolster the potential of this new therapeutic regimen to impede the development of metastatic bone cancer while limiting severe side effects following systemic drug treatment thereby enhancing the care and surgical outcomes in patients.

## 4. Materials and Methods

### 4.1. Lung Cell Line and Lung Cancer-Induced Bone Metastasis Cells

The HCC827 cells purchased from ATCC (CRL-2868™, Cedarlane, Burlington, ON, Canada) are human lung adenocarcinoma-derived cells with a known mutation in the epidermal growth factor receptor (EGFR). Bone metastasis cells were harvested from biopsies obtained from three patients undergoing resection of spinal metastasis of lung origin at the Montreal General Hospital. The study was conducted in accordance with the declaration of Helsinki, and the collection of biopsies was approved by the institutional review board of the Research Institute of the McGill University Health Centre (RI-MUHC)(REB # 2019-4896) titled “Spine Tissue Bank” ” and was undertaken with the consent of each patient with bone metastasis secondary to lung cancer. Biopsy samples which are indicated herein as bone metastasis lung (BML) 1, 3 and 4 were immediately processed after their transfer from the operating room as follows. A portion of each sample was collected onsite by a pathologist and sent to the hospital pathology department for immediate and accurate diagnosis for ongoing surgery. The remainder was immediately delivered to our laboratory, split into several pieces for further processing. 

Fresh biopsies were then either processed for cryosectioning and hematoxylin–eosin (H&E) staining or cancer cell isolation and culture. 

For cryosectioning and H&E staining, samples were cut into 5 mm × 5 mm pieces and placed in cryosectioning molds, covered with optimal cutting temperature (OCT) compound (Fisher—cat 4585, Ottawa, ON, Canada), flash frozen in liquid nitrogen and stored in −80 °C for future use. The frozen specimens were then cut into 5-micron sections using the Leica CM1950 Cryostat (Leica Biosystems Inc., Concord, ON, Canada) and transferred onto glass microscope slides (VWR—cat 48311-703, Mont-Royal, QC, Canada). Sections were stained with Mayer’s Hematoxylin solution (Sigma—cat MHS32, Oakville, ON, Canada) and Eosin Y solution (Sigma—cat HT110116, Oakville, ON, Canada), mounted with permount (Fisher—cat SP15-100, Ottawa, ON, Canada) and covered with cover slips (Fisher—cat 12-545C, Ottawa, ON, Canada). Images of H&E stained slides were taken using the Axioscop 40 light microscope (Zeiss, Thornwood, NY, USA) and then analyzed by a second pathologist for identification of tumor sites. 

For cell isolation and culture, samples were washed with PBS1x (Sigma—cat D5652, Oakville, ON, Canada), cut into ~2 mm × 2 mm pieces and placed in RPMI media [(Gibco, Thermofisher—cat 11835-030, Waltham, MA, USA), 10% fetal bovine serum (FBS) (Gibco, Thermofisher cat—12483-020, Waltham, MA, USA), 1% penicillin streptomycin (PS) antibiotics (Gibco, Thermofisher—cat 15070-063, Waltham, MA, USA), 1% glutamax (Gibco, Thermofisher—cat 35050-061, Waltham, MA, USA)and 1% fungizone (Gibco, Thermofisher—cat 15290-018, Waltham, MA, USA)] containing collagenase (1.5 mg/mL) (Gibco, Thermofisher—cat 17101-015, Waltham, MA, USA) before incubation overnight at 37 °C. Digested cells were centrifuged into a pellet at 1500 rpm for 5 min and then cultured in T75 flasks at 37 °C in supplemented RPMI media (10% FBS, 1% PS, 1% glutamax and 1% fungizone). These isolated cells consisted of a heterogeneous population of bone metastasis cells and bone/stromal cells and cell heterogeneity was verified by the pathologist. All cell cultures were routinely checked to ascertain the presence of mixed population with enriched cancer cells. All in vitro experiments used patient derived cells between 4–5 passages and were continually verified for tumor/stromal cell heterogeneity and further verified by the pathologist.

### 4.2. Proliferation Assays

Cancer cells were seeded at a density of 3000 (HCC827) and 5000 (lung cancer-induced bone metastasis) cells per well in Costar 96 well plates (Fisher Scientific—cat 3882, Canada) with triplicate wells per tested variable in media supplemented with 10% FBS. After 24 h, fresh media with variables were added [vehicle (PBS1x) or zoledronate at concentrations 1, 3 or 10 µM (Sigma—cat SML0223, USA) in low serum-RPMI (1% FBS, 1% PS)]. Experiments were conducted for 7 days with a media change and variable replenishment on day 4. Two metabolic activity-based assays were used to assess proliferation: alamarBlue® (Thermofisher—cat DAL1025, USA) and Vybrant® MTT cell proliferation kit (Thermofisher—cat V13154, USA) as previously described [61]. For both assays, the plate was read using the Tecan Infinite M200 Pro plate reader (Tecan Trading, AG, Männedorf, Zürich, Switzerland) with the following setting: for alamar (excitation wavelength 540 nm and emission wavelength 585 nm) and MTT (absorbance wavelength of 540 nm). Raw data generated from both assays in excel sheets were after background-normalized and analyzed as ratio of zoledronate-treated values over vehicle-treaded values.

### 4.3. Live/Dead Cell Viability Assay

Cells were seeded at 3000 (HCC827) or 5000 (lung cancer-induced bone metastasis) cells per well in 96 well plates (Costar, Fisher Scientific—cat 3882, USA) and incubated at 37 °C for 1 day. The next day, variables were added [Vehicle (PBS1x) or zoledronate concentrations 1, 3 or 10 µM] in RPMI (1% FBS, 1% PS) and experiments were conducted for 7 days with a media change and variable replenishment on day 4. On day 7, well contents were discarded and a PBS1x solution containing 1% calcein AM (green) and 2% ethidium homodimer-1(red) (Themofisher—cat L3224, USA) was added to each well followed by an incubation at 37 °C for 10 min. Pictures of the wells were then taken using an inverted fluorescence microscope Olympus, IX71 (Center Valley, PA, USA) and the cells (green cells for live, red cells for dead) were counted using the ImageJ software (National Institutes of Health, USA; http://imagej.nih.gov/ij).

### 4.4. Caspase 3/7 Activity Assay

Seeding and treatment of HCC827 and lung cancer-induced bone metastasis cells were performed as described above, with some modifications. Cancer cells were treated with only 10 µM of zoledronate and incubated for either 4 or 7 days. Variables replenishment were only made for the 7-day treatment. Caspase 3/7 activity was then assessed using the Cell Meter™ caspase 3/7 activity apoptosis assay Kit (AAT Bioquest—cat 22795, USA) as per the manufacturer’s protocol. Briefly, cells were incubated with the caspase 3/7 assay solution, that contained the fluorogenic caspase substrate/peptide (Ac-DEVD-AMC), at room temperature for 24 h in the dark. Upon incubation, AMC peptides are cleaved by caspase 3 generating strongly fluorescent AMC that are measured on the Tecan Infinite M200 Pro plate reader with the following setting (excitation 350 nm and emission 450 nm). To account for the number of cells available in wells following treatment and assessment of caspase activity, Millipore© BrdU cell proliferation Kit, a DNA synthesis-based assay (Millipore—cat 2750, USA) was used following the manufacturer’s protocol (absorbance wavelength 450 nm). Caspase and BrdU raw data were expressed as ratio of zoledronate-treated over vehicle-treaded values. The caspase ratios were then normalized to BrdU ratios to generate the caspase activity of lung cancer and bone metastasis cells. 

### 4.5. Transwell Cell Migration Assay

Cells were seeded at a density of 20,000 cells per Falcon™ cell culture insert (8 µm pore size; VWR, Falcon—cat 353097, Mont-Royal, QC, Canada) with 10% FBS, 1% PS RPMI media. Chambers were placed inside a 24 well plate (Fisher Scientific—cat 83.3922.300, Canada) filled with 10% FBS, 1% PS media. The next day, media was discarded from both chambers and wells and then replaced with low serum-RPMI media (1% FBS, 1% PS in chamber and 2% FBS, 1% PS in well) containing the variables [(vehicle (PBS1x) or zoledronate 1, 3 or 10 µM]. Media containing variables were discarded and replenished on day 4. On day 7, chambers were washed with PBS1x, fixed with 4% paraformaldehyde (Thermofisher—cat 28908, USA), and cells in the upper part of the membrane were wiped away using cotton swabs. Membranes were then cut out of the chambers, mounted onto microscope slides and stained with DAPI (Sigma—cat F6057, USA). The membranes were imaged using an inverted fluorescence microscope (Olympus, IX71) and the cells were counted using the ImageJ software.

### 4.6. 3D Matrix Invasion Assay

Cells were seeded at 5000 cells per well in round bottom clear 96 well plates (USA, Costar, Fisher Scientific—cat 7007) suspended in RPMI 10% FBS, 1% PS containing 1:10 dilution of spheroid formation buffer (Thermofisher—cat 3500-096-K, USA). The plate was then centrifuged 200× *g* at 20 °C for 3 min before incubation at 37 °C for 3 days. Gel matrix (Thermofisher—cat 3500-096-K, USA) was then added to each well and the plate was centrifuged 300× *g* at 4 °C for 5 min before incubation at 37 °C for 1 h. Variables were then added in RPMI (1%FBS, 1% PS) to each well [Vehicle (PBS1x) or, zoledronate 1, 3, 10 µM] and the plate was re-incubated at 37 °C for 1, 4, 7 and 12 days. Images were captured using a Canon powershot camera A640 with a Soligor adaptor 426,126 and an inverted light microscope Axiovert 40 C (Zeiss, Thornwood, NY, USA) at 10× magnification. Area of spheroid invasion was measured using the ImageJ software.

### 4.7. Statistics

Data from each experiment was transferred to Excel datasheet (Microsoft Office 2016). Statistical analyses were performed using R v.3.4.1 (The R Core Team, 2016) and R studio Software (USA, version 1.1.453). All data are expressed as the mean ± SD. Data normality was tested using Shapiro-Wilk test. Comparisons between groups were made by one-way ANOVA and Tukey post-hoc tests or independent t-tests at a 95% confidence level. When heteroscedasticity was present, White’s adjust was performed. *p* values < 0.05 were considered as statistically significant.

## Supplementary Materials

The following are available online at https://www.mdpi.com/2077-0383/8/8/1212/s1, Figure S1: Spheroid invasion assay of HCC827 and lung cancer-induced bone metastasis cells treated with vehicle (PBS1x) or Zol 1 µM, 3 µM and 10 µM for 1 and 4 days in 1% serum conditions.
