# Supplementary material for: Anti-Tumor Effects of Low Dose Zoledronate on Lung Cancer-Induced Spine Metastasis
Source: J Clin Med. 2019 Aug 14;8(8):1212. doi: 10.3390/jcm8081212 (PMC6722631; doi:10.3390/jcm8081212)
Supplement: Supplementary file 1 [file jcm-08-01212-s001.pdf]

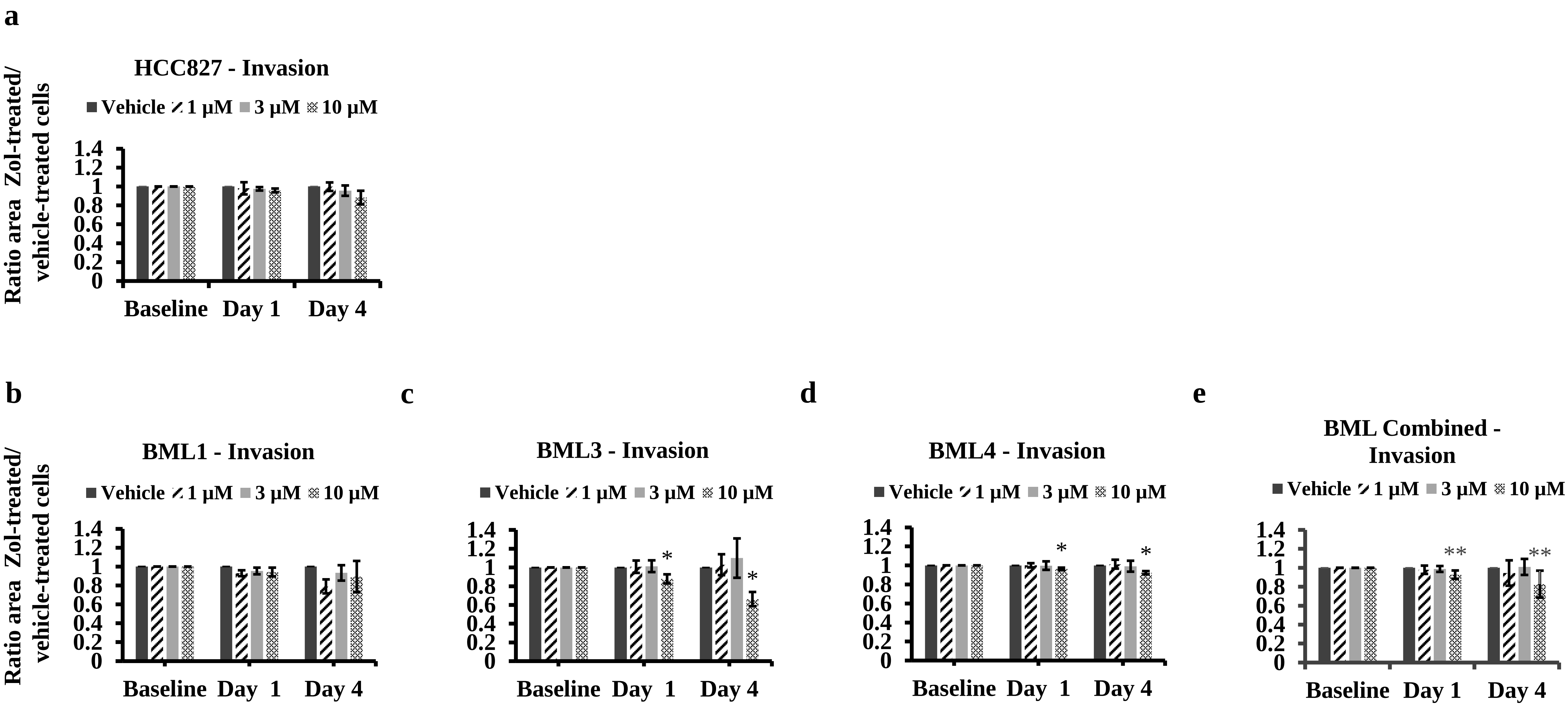
a
HCC827 - Invasion
Vehicle
1 µM
3 µM
10 µM
Ratio area Zol-treated/ vehicle-treated cells
1.4
1.2
1
0.8
0.6
0.4
0.2
0
Baseline
Day 1
Day 4
b
BML1 - Invasion
Vehicle
1 µM
3 µM
10 µM
Ratio area Zol-treated/ vehicle-treated cells
1.4
1.2
1
0.8
0.6
0.4
0.2
0
Baseline
Day 1
Day 4
c
BML3 - Invasion
Vehicle
1 µM
3 µM
10 µM
1.4
1.2
1
0.8
0.6
0.4
0.2
0
*
*
Baseline
Day 1
Day 4
d
BML4 - Invasion
Vehicle
1 µM
3 µM
10 µM
1.4
1.2
1
0.8
0.6
0.4
0.2
0
*
*
Baseline
Day 1
Day 4
e
BML Combined - Invasion
Vehicle
1 µM
3 µM
10 µM
1.4
1.2
1
0.8
0.6
0.4
0.2
0
**
**
Baseline
Day 1
Day 4

Figure S1. Spheroid invasion assay of HCC827 cells and lung-induced bone metastasis cells treated with vehicle (PBS1x) or Zol 1 μM, 3μM and 10 μM for 1 and 4 days in 1% serum conditions. Histograms for growth in the matrix for HCC827 (a) and lung-induced bone metastasis cells (b, c, d) showing the ratio of drug-treated cells divided by vehicle-treated cells (PBS1x) ± SD for three independent experiments. e) combined ratio for the three cell types of lung-induced bone metastasis cells are presented. Each condition (drug-treated cells or vehicle-treated cells) on each day was normalized to day 0 and then to vehicle. Significantly different from control $^{*}p < 0.05$, $^{**}p < 0.01$.
